# *Parrotia persica* Yellow and Amber Leaves’ Lipophilic Phytochemicals Obtained by Supercritical Carbon Dioxide Extraction

**DOI:** 10.3390/molecules27165237

**Published:** 2022-08-17

**Authors:** Nina Djapic

**Affiliations:** Technical Faculty “Mihajlo Pupin”, University of Novi Sad, Djure Djakovica bb, 23000 Zrenjanin, Serbia; nidjapic@gmail.com or nina.djapic@tfzr.rs

**Keywords:** *Parrotia persica*, leaves, α-tocopherol, SC CO_2_

## Abstract

Supercritical carbon dioxide extraction was used for the extraction of *Parrotia persica* yellow and amber leaves. The lipophilic phytochemicals present in the analyzed leaves were as follows: neophytadiene, hexahydrofarnesyl acetone, octadecanal, 1-octadecanol, phytol, squalene and α-tocopherol. α-cadinol was present in yellow and β-sitosterol in amber leaves. The Box–Behnken design was used for the optimization of pressure, temperature and CO_2_ flow rate and response surface methodology for the total extraction yield and α-tocopherol relative amount. The total extraction yield was 1.62% for yellow and 1.52% for amber leaves. The α-tocopherol relative amount was 80.03 mg per 100 g of dry plant material for yellow leaves and 315.30 mg per 100 g of dry plant material for amber leaves. The effects of temperature and CO_2_ flow rate were found to have a significant influence on the total extraction yield for both plant materials analyzed. The effects of pressure and temperature significantly influenced the α-tocopherol relative amount in both plant materials used. The optimum extraction conditions for the total extraction yield were 30 MPa, 40 °C and 3 kg·h^−1^ CO_2_ flow rate for both plant samples. In the case of the α-tocopherol relative amount, the optimum temperature was 40 °C, while the pressure and CO_2_ flow rate were slightly different. The predicted values matched well with the experimental values for the total extraction yield and α-tocopherol relative amount in all plant materials used for the experiment.

## 1. Introduction

The autumn brings the finest displays, with various yellow, gold, orange, coppery red, crimson, cream and amber exfoliates on thin leaves plates to reveal the autumnal and winter leaf colors. For several weeks in late autumn, every *Parrotia persica* (DC) C. A. Mey., Hamamelidaceae leaf turns to a different color, including red, crimson, orange and yellow. In winter, the *P. persica* leaves turn to cream and amber. *P. persica* grows naturally on the Caucasus and in Caspian forests [1]. Aerial parts of *P. persica* (leaves and stems) were extracted with methanol, the chemical constituents present in the obtained extract were purified by column chromatography and the flavonoids were determined by different spectroscopic techniques. [2]. *P. persica* leaves were extracted with various organic solvents and the extracts obtained were evaluated for antibacterial activity [3].

In autumn, with the beginning of leaf senescence, chlorophyll molecules rupture. The hydrolysis of chlorophyll reveals the phytol and chlorophyllide molecule [4]. One pathway for tocopherol biosynthesis uses phytol derived from chlorophyll [4,5]. Tocopherols are compounds known for their antioxidant activity [6]. The tocopherols are present in soybean and wheat germ oils [7]. The methods for obtaining tocopherols include extraction from natural sources or chemical synthesis [8]. On the market, the mixture of tocopherols is available in vegetable oils [8]. Tocopherols are present in different vegetable oils [9].

Supercritical carbon dioxide (SC CO_2_) extraction has higher extraction rates, efficiencies and selectivity compared to extractions using solvents [10]. SC CO_2_ extraction has been used for the recovery of tocopherols from soybean oil deodorizer and tocopherols were concentrated from 11% to 13% [11]. The operational conditions were from 40 °C to 80 °C and from 90 to 170 bar [11]. It was concluded that the yield of the process reduces with the increase in pressure at constant temperature [11]. Various SC CO_2_ extractions were carried out or simulated for canola, lampante olive oil, sunflower oil deodorizer distillate and esterified olive oil deodorizer distillates [12,13]. The recovery of tocopherols from the deodorized distillate of the vegetable oil using SC CO_2_ has been reported [14,15]. The effective separation of linoleic acid, stigmasterol and squalene from tocopherols was investigated [14]. At 40 °C and 90 bars, the separation between tocopherols and linoleic acid had an efficiency of 93.1% and the concentration factor showed that the tocopherols were completely separated from the fatty acids [14]. The optimal conditions for separation between tocopherols and squalene was at 40 °C and 350 bar [14]. The separation between tocopherols and stigmasterol was at 40 °C and 250 bars, with the efficiency of 99.0% and concentration factor of 103.6 [14]. At the same operating conditions, tocopherols remained with squalene because the separation was not efficient at low pressures [14]. The recovery of tocopherols, contained in the esterified soybean sludge, can be up to a maximum of 40 wt% [15]. SC CO_2_ has been used for the extraction of tocopherol concentrates from olive tree leaves [16]. The tocopherol extraction rates were determined in the function of pressure (25–45 MPa), particle size (0.25–1.5 mm), solvent flow (0.5–1.5 SL·min^−1^) and temperature (40–60 °C) [16]. At 40 °C, pressure of 25 MPa, solvent flow rate of 1 SL·min^−1^ and particle size of 1.5 mm, the tocopherol concentration in the extract was 74.5% [16]. The Soxlet extraction using hexane showed no tocopherol content, which was most probably due to thermal and oxidative degradation [16]. Tocopherol extraction from the yellow and amber *P. persica* leaves has not been performed, up to now. The main goals of the present study were as follows: to find the lipophilic phytochemical profile of yellow and amber *P. persica* leaf extracts obtained by SC CO_2_, to investigate the influence of process parameters on SC CO_2_ extraction and to find the optimal extraction conditions related to the extraction yield and α-tocopherol amount.

## 2. Results

### 2.1. Design of the Experiments

The Box–Behnken design (BBD) from response surfacy methodology (RSM) was used to optimize the three most important operating SC CO_2_ extraction variables in order to achieve the highest total extraction yield and the highest relative amount of the α-tocopherol (Table 1) [17,18,19]. The three independent variables were extraction pressure (*X*_1_), extraction temperature (*X*_2_) and CO_2_ flow rate (*X*_3_) for uncoded variable levels. The settings for these independent variables are depicted in Table 1. The analysis was performed using Minitab, LLC, 2021 (Chicago, IL, USA).

### 2.2. Lipophilic Phytochemicals of Yellow and Amber P. persica Leaves

The phytochemicals present in the *P. persica* yellow leaf SC CO_2_ extracts are depicted in Table 2.

The phytochemicals present in amber *P. persica* SC CO_2_ extracts are depicted in Table 3.

α-tocopherol was chosen for the determination of its relative amount in the samples obtained by SC CO_2_ extraction to investigate the changes in its biosynthesis from yellow to amber leaves.

### 2.3. The Analysis of Variance

The effect of linear, quadratic and interaction coefficients on the response was analyzed for significance by the analysis of variance (ANOVA). The degree of significance for each factor is represented by the *p*-values. For the confidence level of 0.95, the *p* < 0.05 has a significant influence on the process. The analysis of variance for the total extraction yield of *P. persica* yellow and amber leaves is depicted in Table 4.

The regression coefficients from the RSM were estimated for the full quadratic BBD extraction yield of *P. persica* yellow leaves with the Equation (1), which is as follows:

Yield (%) = −0.841 + 0.053 *X*_1_ + 0.041 *X*_2_ + 0.607 *X*_3_ − 0.00077 *X*_1_·*X*_1_ − 0.00025 *X*_2_·*X*_2_ − 0.1146 *X*_3_·*X*_3_ − 0.00007 *X*_1_·*X*_2_ + 0.01 *X*_1_·*X*_3_ − 0.00375 *X*_2_·*X*_3_ (1)

**Figure 1:**
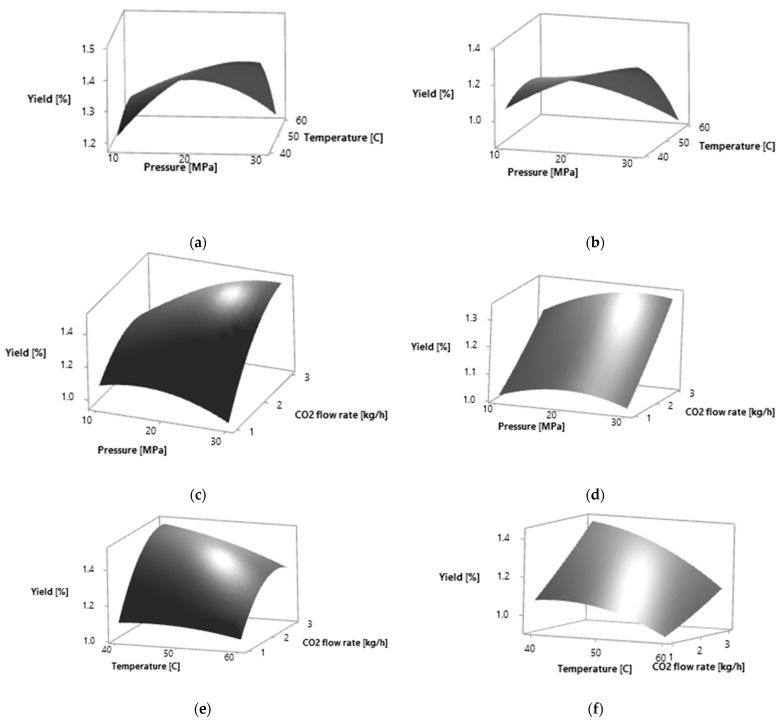
Response surfaces for effects of two independent variables on the extraction yield of *P. persica* leaves by SC CO_2_ extraction: (**a**) pressure (MPa) and temperature (°C) for yellow leaves; (**b**) pressure (MPa) and temperature (°C) for amber leaves; (**c**) pressure (MPa) and CO_2_ flow rate (kg·h^−1^) for yellow leaves; (**d**) pressure (MPa) and CO_2_ flow rate (kg·h^−1^) for amber leaves; (**e**) temperature (°C) and CO_2_ flow rate (kg·h^−1^) for yellow leaves; (**f**) temperature (°C) and CO_2_ flow rate (kg·h^−1^) for amber leaves.

The regression model for the total extraction yield was highly significant according to the *p*-value with the satisfactory coefficients of determination. The coefficient of determination *R*^2^ was 0.9486, while the *R*^2^ adjusted was 0.8562. The obtained values were used for the construction of the three-dimensional graphs of the RSM (Figure 1).

From the Minitab^®^ software response optimizer, the optimal parameters for the maximum total extraction yield were defined in terms of 30 MPa, 40 °C and 3.0 kg·h^−1^. Three extractions under optimal conditions were carried out to validate the mathematical model developed for the maximum extraction yield of yellow *P. persica* leaves. Under the optimal conditions, the experimental value for the extraction yield was 1.62%. The predicted data were experimentally confirmed with the deviation within ±5%.

For the extraction yield of *P. persica* amber leaves, the regression coefficients from the RSM were estimated for the full quadratic BBD with Equation (2).

Yield (%) = −1.57 + 0.074 *X*_1_ + 0.076 *X*_2_ + 0.252 *X*_3_ − 0.00043 *X*_1_·*X*_1_ − 0.00056 *X*_2_·*X*_2_ + 0.0092 *X*_3_·*X*_3_ − 0.00117 *X*_1_·*X*_2_ + 0.0022 *X*_1_·*X*_3_ − 0.004 *X*_2_·*X*_3_ (2)

The coefficient of determination R^2^ was 0.9155, while the R^2^ adjusted was 0.7634. The optimal parameters calculated for the maximum total extraction yield were the same as for the extraction yield of yellow *P. persica* leaves. Three extractions under optimal conditions were carried out. Under the optimal conditions, the experimental value for the extraction yield was 1.52%. The deviation was within ±5% between the mathematically predicted data and experimental data.

The obtained values were used for the construction of the three-dimensional graphs of the RSM (Figure 1). 

The analysis of variance for the α-tocopherol relative amount of *P. persica* yellow and amber leaves is depicted in Table 5.

The regression coefficients from the RSM were estimated for the full quadratic BBD α-tocopherol relative amount of *P. persica* yellow leaves with Equation (3).

α-Tocopherol (mg·100 g^−1^) = 309.6 + 2.82 *X*_1_ − 10.13 *X*_2_ + 26.0 *X*_3_ − 0.055 *X*_1_·*X*_1_ + 0.082 *X*_2_·*X*_2_ − 2.78 *X*_3_·*X*_3_ − 0.008 *X*_1_·*X*_2_ − 0.254 *X*_1_·*X*_3_ − 0.178 *X*_2_·*X*_3_ (3)

The coefficient of determination R^2^ was 0.9918, while the R^2^ adjusted was 0.977 for the model summary. The solutions for the process parameters to obtain the maximum α-tocopherol relative amount included pressure of 16.46 MPa, temperature of 40 °C and CO_2_ flow rate of 2.65 kg·h^−1^. The solution process parameters were applied on three extractions and the α-tocopherol relative amount of 80.03 mg per 100 g of the dry plant material with the deviation within ±5% was obtained.

**Figure 2:**
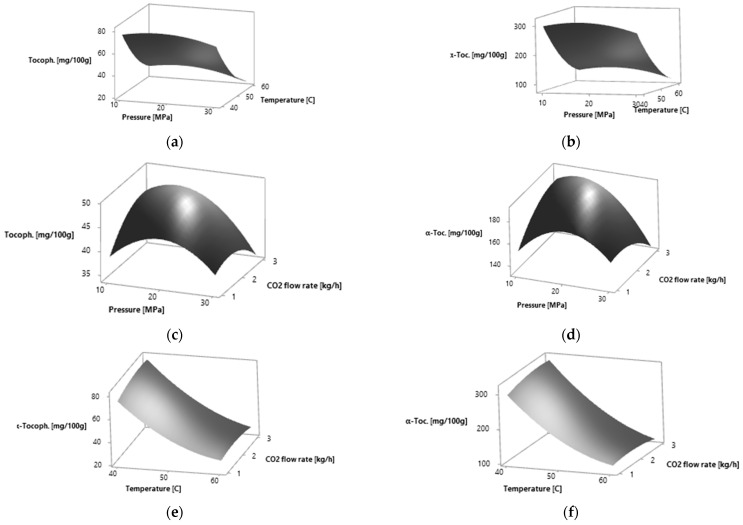
Response surfaces for effects of two independent variables on the α-tocopherol relative amount of *P. persica* leaves by SC CO_2_ extraction: (**a**) pressure (MPa) and temperature (°C) for yellow leaves; (**b**) pressure (MPa) and temperature (°C) for amber leaves; (**c**) pressure (MPa) and CO_2_ flow rate (kg·h^−1^) for yellow leaves; (**d**) pressure (MPa) and CO_2_ flow rate (kg·h^−1^) for amber leaves; (**e**) temperature (°C) and CO_2_ flow rate (kg·h^−1^) for yellow leaves; (**f**) temperature (°C) and CO_2_ flow rate (kg·h^−1^) for amber leaves.

The obtained values were used for the construction of the three-dimensional graphs of the RSM (Figure 2).

The influence of pressure, temperature and CO_2_ flow rate on the relative amount of α-tocopherol in amber *P. persica* leaf extracts was measured by the analysis of variance (Table 5). The regression coefficients from the RSM for the α-tocopherol relative amount were estimated for the full quadratic BBD model for the noncoded variables with Equation (4).

α-Tocopherol (mg·100 g^−1^) = 1180 + 11.55 *X*_1_ − 38.15 X_2_ + 92.4 *X*_3_ − 0.2276 *X*_1_ *X*_1_ + 0.302 *X*_2_·*X*_2_ − 8.68 *X*_3_·*X*_3_ − 0.0204 *X*_1_·*X*_2_ − 1.271 *X*_1_·*X*_3_ − 0.599 *X*_2_·*X*_3_ (4)

The coefficients of determination *R*^2^ and *R*^2^ adjusted were 0.9915 and 0.9762, respectively. The solutions for the process parameters to obtain the maximum α-tocopherol relative amount from the *P. persica* amber leaves included pressure of 15.86 MPa, temperature of 40 °C and CO_2_ flow rate of 2.78 kg·h^−1^. The solution process parameters were applied on three extractions and the α-tocopherol relative amount of 315.30 mg per 100 g of the dry plant material was obtained. The calculated data were experimentally confirmed with a deviation of ±5%.

The obtained values were used for the construction of the three-dimensional graphs of the RSM (Figure 2).

## 3. Discussion

The phytochemical compounds present in the yellow and amber leaves were as follows: neophytadiene, hexahydrofarnesyl acetone, octadecanal, 1-octadecanol, phytol, squalene and α-tocopherol (Table 2 and Table 3). The yellow leaves contained α-cadinol, while in the amber leaves, it was not detected. The β-sitosterol was present in amber leaves.

The effect of pressure, temperature and CO_2_ flow rate was investigated using the BBD with three levels for each factor. The BBD consisted of fifteen experiments (Table 1). The average particle size, the mass of the plant material in the extractor and the extraction time were kept constant during all experiments. The extraction time was kept constant, due to the results obtained where extractions longer than 90 min did not significantly increase the extraction yield. The extraction yield varied from 0.94% (30 MPa, 50 °C and CO_2_ flow rate 1 kg·h^−1^) to 1.55% (30 MPa, 50 °C and CO_2_ flow rate 3 kg·h^−1^) for the yellow *P. persica* leaves (Table 1). For the amber *P. persica* leaves, the extraction yield was 0.82% (30 MPa, 60 °C and CO_2_ flow rate 2 kg·h^−1^), while the highest extraction yield was 1.39% (20 MPa, 40 °C and CO_2_ flow rate 3 kg·h^−1^) (Table 1). The interaction coefficient pressure and CO_2_ flow rate for yellow leaves and pressure and temperature for amber leaves had a significant influence on the extraction yield. The quadratic interaction coefficients for CO_2_ flow rate showed the statistically significant influence on the extraction yield from the yellow leaves. The second order polynomial models were used to express the total extraction yield in the function of independent variables and are expressed by Equation (1) for the yellow leaves and Equation (2) for amber leaves. From Equation (1), the quadratic terms of pressure (*X*_1_^2^) and temperature (*X*_2_^2^) were statistically insignificant and this reflection can be concluded from the values of the regression coefficients. Interactions between pressure and temperature (*X*_1_·*X*_2_) and interactions between temperature and CO_2_ flow rate (*X*_2_·*X*_3_) can be removed from the model because they have no significant effect on the extraction yield. In case of Equation (2), the two-way and quadratic interactions have no significant effect on the extraction yield. The RSM analysis confirmed that the temperature and CO_2_ flow rate are highly statistically significant model parameters that influence the process.

The calculated optimal conditions for obtaining the maximum extraction yield were, for both plant materials, 30 MPa, 40 °C and flow rate of 3 kg·h^−1^. The higher selectivity of the total extraction yield was obtained at lower temperatures and higher flow rates. On the selected surface plots, it can be observed that the total extraction yield increases with the increase in the CO_2_ flow rate (Figure 1). With the increase in pressure and CO_2_ flow rate, the total extraction yield increases in both plant materials analyzed. The total extraction yield is the highest at lower temperatures.

Three extractions under optimal conditions were carried out to validate the developed mathematical model for the total extraction yield. The obtained experimental data are in the range of the predicted values and are within the limits set by the relevant confidence intervals with the deviation of ±5%. This confirms the acceptable accuracy of the assumed mathematical models and reliability of the BBD.

The highest relative amount of α-tocopherol in yellow leaves was 81.67 mg per 100 g (20 MPa, 40 °C and CO_2_ flow rate 3 kg·h^−1^), while the lowest relative amount was 22.65 mg per 100 g (30 MPa, 60 °C and CO_2_ flow rate 2 kg·h^−1^) (Table 2). In amber leaves, the highest α-tocopherol relative amount was 321.68 mg per 100 g (20 MPa, 40 °C and CO_2_ flow rate 3 kg·h^−1^), while the lowest relative amount found was 90.27 mg per 100 g (30 MPa, 60 °C and CO_2_ flow rate 2 kg·h^−1^) (Table 3).

In the case of the α-tocopherol relative amount, the response variables were positively correlated with pressure (*X*_1_) and temperature (*X*_2_) and the quadratic terms of pressure (*X*_1_^2^) and quadratic terms of temperature (*X*_2_^2^) were regarded as statistically significant. The linear and quadratic terms of CO_2_ flow rate (*X*_3_) and (*X*_1_^2^) turned out to be statistically insignificant (*p* > 0.05). All the two-way interactions were statistically insignificant. It can be concluded from the statistical analysis of regression coefficients that the α-tocopherol relative amount depends on the pressure (*X*_1_) and temperature (*X*_3_) for both plant material analyzed (Table 5).

The effects of process parameters on the studied response variables, the α-tocopherol relative amount, were drawn in the form of response surface figures (Figure 2). The total α-tocopherol relative amount increases when increasing the pressure to 16.54 MPa for yellow leaves and to 15.85 MPa for amber leaves and then it decreases. The highest α-tocopherol relative amount was obtained at 40 °C. At the CO_2_ flow rate of 2.65 kg·h^−1^ for yellow leaves and 2.77 kg·h^−1^ for amber leaves, the highest α-tocopherol relative amount was obtained. A further increase in the CO_2_ flow rate led to the decrease in α-tocopherol relative amount.

Three extractions under optimal conditions were carried out to validate the developed mathematical model for the α-tocopherol relative amount. The obtained experimental data were as follows: 80.03 mg of α-tocopherol per 100 g of the dry plant material for the yellow leaves and 315.3 mg of α-tocopherol per 100 g of the dry plant material for the amber leaves. The results obtained were in the range of the predicted values and relevant confidence intervals with the deviation of ±5%. This permitted the acceptable accuracy of the assumed mathematical models.

The parameters that influenced the α-tocopherol extraction yield from the various leaves investigated were not uniform. The highest α-tocopherol extraction yield, in the case of olive leaves, was 10.10 mg per 100 g of leaves at 25 MPa, 40 °C, CO_2_ flow rate of 1 SL·min^−1^, particle diameter 1.5 mm and extraction time of 120 min [16]. Under the same parameters, only the extraction time was 60 min, the α-tocopherol extraction yield was 6.94 mg per 100 g of leaves [16]. In case of *Eugenia involucrata,* the highest α-tocopherol extraction yield was 68.27 mg per 100 g of leaves when the pressure was 20 MPa, temperature was 60 °C and the CO_2_ flow rate was 4 mL·min^−1^ [20]. The α-tocopherol extraction yield in the case of *Pandanus odorus* leaves increased with the increase in pressure and decreased when increasing the temperature [21]. At a pressure of 200 kg·cm^−2^ and at 40 °C, the yield was ~300 ppm after 3 h of extraction, while at the same temperature, but at pressure of 80 kg·cm^−2^, the α-tocopherol extraction yield was 134 ppm [21]. In *Vitis vinifera* leaves, α-tocopherol was only detected when the extraction parameters were as follows: pressure of 30 MPa, temperature of 40 °C, particle diameter of 10 mm and CO_2_ flow rate of 80 kg·m^−3^ [22]. The *P. persica* yellow and amber leaves had the highest α-tocopherol extraction yield at the pressure of 20 MPa, temperature of 40 °C and CO_2_ flow rate of 3 kg·h^−1^. In the case of *E. involucrate,* the temperature was the highest and obtained the highest α-tocopherol extraction yield compared to olive, *P. odorus*, *V. vinifera* and *P. persica* leaves. Yellow *P*. *persica* leaves have a higher α-tocopherol extraction yield compared to *E. involucrate*. The highest α-tocopherol extraction yield was demonstrated by amber *P. persica* leaves.

## 4. Materials and Methods

### 4.1. Chemicals

The CO_2_ used for extractions was 99.97% (Messer, Tehnogas AD, Rakovica, Serbia). The calibration curves were prepared using α-tocopherol (Dr. Ehrenstorfer, Augsburg, Germany). The *n*-hexane was provided from Merck, Darmstadt, Germany.

### 4.2. Plant Material

*P. persica* yellow leaves were collected at the beginning of October and amber leaves were collected in late October 2021 in the Futoski Park, Novi Sad, Serbia (45°14′58.8″ N, 19°49′38.3″ E). Leaves were air dried at 16 °C for three days. Dried leaves were grounded by a laboratory mill. After drying, the water content of grounded leaves was determined according to AOAC Official Method 925.40 and was 10.37 ± 0.12% for grounded yellow leaves and 8.54 ± 0.10% for the amber grounded leaves. Prior to extraction, the grounded plant material was sieved for 15 min using the vertical vibratory sieve shaker (Labortechnik GmbH, Ilmenau, Germany). The plant material powder size distribution was determined using a nest of five sieves with aperture sizes 0.1, 0.2, 0.315, 0.4 and 0.5 mm. The mass of fragments remaining on each sieve was used to calculate the distribution of fragments, which were normalized in respect to the total mass. The Rosin–Rammler (RR) distribution was used for the calculation of the sieve analysis results [23]. The average particle size was determined and was 0.378 ± 0.09 mm for yellow grounded plant material and 0.367 ± 0.08 mm for the amber grounded plant material. All measurements were performed in triplicate. 

### 4.3. Extraction Procedure

The extractions were carried out on the laboratory-made SC CO_2_ system, consisting of a CO_2_ reservoir, cooling bath (ethylene-glycol/ethanol), air compressor, air-driven CO_2_ pump (Haskel^®^ MS-71), heating bath, extraction cell, separator vessel and flow meter (Matheson FM-1050, E800). Temperature was controlled using a proportional-integral-derivative (PID) controller. Pressure controllers included two manometers for the pressure control in the extraction cell and one for the pressure control in the separator (WIKA, model 212.20). The plant material powder (50 g) was poured into the extractor vessel. The extract was collected in a separator with the glass tube with previously determined glass tubes mass. The extractions were carried out at different conditions determined by the BBD [17,18,19]. The mass of the plant material in the extractor, grounded plant material particle size and the extraction time (90 min) were kept constant during the experiment. Extraction longer than 90 min did not increase the extraction yield significantly, based on the experiments performed. The obtained extract yields were determined by a balance with precision of ±0.0001 g. The extraction yields are expressed in percentages (grams of extract per 100 g of the sample). The extracts obtained were kept at 4–6 °C until GC–MS analysis. 

### 4.4. Experimental Design

The SC CO_2_ extraction optimal process conditions were calculated using the BBD and the response surface methodology (RSM) [17]. The three coded variables were as follows: the extraction pressure (*X*_1_), temperature (*X*_2_) and CO_2_ flow rate (*X*_3_). The uncoded variables were studied in order to optimize the extraction process to obtain a higher total extraction yield and α-tocopherol relative amount. The settings for the coded variables are depicted in Table 6.

Experimental data were fitted with the second-order response surface model with Equation (5).

(5) $$Y=\beta_{0}+\sum_{i=1}^{k}\beta_{i}X_{i}+\sum_{i=1}^{k}\beta_{ii}X_{i}^{2}+\sum_{\begin{array}{l} i=1\\ i<j \end{array}}^{k-1}\sum_{j=2}^{k}\beta_{ij}X_{i}X_{j}$$

where *Y* is the investigated response, *β*_0_, *β*_*i*_, *β*_*ii*_, *β*_*ij*_ are the constant coefficients of intercept, linear, quadratic and interaction terms, respectively; *X_i_* and *X_j_* are the input variables. The analysis was performed using Minitab, LLC, 2021. The analysis of variance was used to evaluate the fitted model quality. The statistical difference test was based on the total error criteria with a confidence level of 95%.

### 4.5. GC–MS Analysis

The extracted samples were dissolved in *n*-hexane. The GC–MS analyses were carried out on an Agilent 7890A GC fitted with a mass selective detector 5975C (Agilent Technologies, Palo Alto, CA, USA). The capillary column was HP-5MS (5% phenyl-methyl polysiloxane, 30 m × 250 μm × 0.25 μm). The carrier gas was He at 1 mL·min^−1^. The injection port temperature was 250 °C. The HP-5MS temperature was set at 70 °C isothermal for 2 min and then increased to 200 °C·min^−1^ and held isothermal for 18 min. The split ratio was 1:50, ionization voltage 70 eV and ion source temperature was 230 °C. The mass scan range was 30–300 mass units. The injected sample volume was 1 μL. The identification of components was carried out based on computer matching with the NIST 2008 MS library. The percentage composition was calculated from the GC peak areas using the normalization method. The quantification of compounds was provided using calibration curves. The standard compound was α-tocopherol. The standard compound was dissolved in *n*-hexane to prepare six different concentrations of α-tocopherol. The R^2^ for the calibration curve was 0.999. All analyses were performed in triplicate. 

## 5. Conclusions

The SC CO_2_ yellow and amber leaf extracts’ lipophilic phytochemicals were almost the same. The relative amount of lipophilic phytochemicals was influenced by the extraction parameters applied. The main difference observed was that the relative amount of phytol in yellow leaves was higher than in amber leaves. The relative amount of α-tocopherol in the yellow leaves was lower than in the amber leaves. The explanation is that phytol feeds one α-tocopherol biosynthetic pathway. The present study provides the data on the optimization of α-tocopherol SC CO_2_ extraction. The optimal temperature was 40 °C. Further studies can include optimizations at temperatures lower than 40 °C.

## Figures and Tables

**Table 1:** The RSM analysis and observed responses for the *P. persica* yellow and amber leaves’ total extraction yield (%).

No.	Pressure (MPa)	Temperature (°C)	CO_2_ Flow Rate (kg·h^−1^)	Extraction Yield Yellow Leaves (%)	Extraction Yield Amber Leaves (%)
1.	10 (−1)	40 (−1)	2 (0)	1.21	1.12
2.	30 (+1)	40 (−1)	2 (0)	1.43	1.35
3.	10 (−1)	60 (+1)	2 (0)	1.27	1.06
4.	30 (+1)	60 (+1)	2 (0)	1.19	0.82
5.	10 (−1)	50 (0)	1 (−1)	1.02	0.99
6.	30 (+1)	50 (0)	1 (−1)	0.94	1.04
7.	10 (−1)	50 (0)	3 (+1)	1.23	1.22
8.	30 (+1)	50 (0)	3 (+1)	1.55	1.36
9.	20 (0)	40 (−1)	1 (−1)	1.16	1.05
10.	20 (0)	60 (+1)	1 (−1)	1.08	0.97
11.	20 (0)	40 (−1)	3 (+1)	1.47	1.39
12.	20 (0)	60 (+1)	3 (+1)	1.24	1.15
13.	20 (0)	50 (0)	2 (0)	1.38	1.09
14.	20 (0)	50 (0)	2 (0)	1.34	1.20
15.	20 (0)	50 (0)	2 (0)	1.41	1.27

**Table 2:** The relative amount (in mg α-tocopherol equivalents per 100 g of dry leaves) of the phytochemicals in the SC CO_2_ extracts of *P. persica* yellow leaves.

No.	α-Cadinol	Neophytadiene	Hexahydrofarnesyl Acetone	Octadecanal	1-Octadecanol	Phytol	Squalene	α-Tocopherol
1.	0.32	32.61	1.06	20.34	1.97	31.72	90.61	75.86
2.	0.36	29.93	0.41	15.87	2.23	28.14	82.07	73.64
3.	-	21.77	0.27	14.46	1.52	20.18	49.26	28.03
4.	-	57.34	0.64	18.24	2.91	47.08	64.53	22.65
5.	-	16.17	0.16	10.07	0.83	21.39	35.67	41.76
6.	-	19.60	0.11	8.10	0.77	12.56	19.68	38.43
7.	0.15	38.12	0.53	13.62	2.06	26.68	52.11	44.79
8.	0.22	71.95	1.02	25.83	4.03	68.14	98.25	31.29
9.	0.08	29.58	0.08	1.75	0.18	3.86	103.34	72.36
10.	-	27.73	0.39	10.91	1.59	36.04	44.19	27.52
11.	0.38	44.41	0.94	16.02	2.23	42.46	68.63	81.67
12.	-	34.86	0.46	12.54	2.07	35.04	53.27	29.71
13.	0.19	31.05	0.67	16.40	1.82	54.67	66.33	46.15
14.	0.23	26.57	0.73	15.37	1.91	51.92	70.28	47.82
15.	0.28	33.25	0.79	17.23	2.06	48.21	68.71	48.23

**Table 3:** The relative amount (in mg α-tocopherol equivalents per 100 g of dry leaves) of the phytochemicals in the SC CO_2_ extracts of *P. persica* amber leaves.

No.	Neophytadiene	Hexahydrofarnesyl Acetone	Octadecanal	1-Octadecanol	Phytol	Squalene	α-Tocopherol	β-Sitosterol
1.	11.48	4.25	15.81	5.68	15.36	44.28	295.24	-
2.	9.21	1.17	12.37	9.34	17.83	39.97	283.56	-
3.	7.35	0.83	11.78	4.26	10.37	24.16	110.12	1.32
4.	18.64	1.60	15.93	8.71	23.81	31.36	90.27	-
5.	5.28	0.48	7.86	2.33	11.29	17.54	163.84	1.17
6.	6.43	0.26	6.49	1.97	5.76	9.71	160.39	0.90
7.	12.59	1.38	11.52	6.18	12.63	25.68	176.81	1.46
8.	23.28	2.97	21.07	11.96	33.40	57.42	122.53	-
9.	9.73	0.19	1.16	0.47	1.76	51.27	287.94	-
10.	8.98	0.86	8.74	4.39	17.92	21.58	108.07	-
11.	14.61	2.73	12.85	6.26	21.08	32.69	321.68	-
12.	11.30	1.16	10.42	6.03	16.75	27.82	117.84	-
13.	10.07	1.72	13.23	5.99	27.09	31.23	184.60	1.83
14.	8.48	2.05	14.57	5.05	25.14	34.80	190.04	1.64.
15.	11.26	2.38	15.09	4.74	27.33	33.07	187.36	1.98

**Table 4:** The analysis of variance for the total extraction yield of *P. persica* yellow leaves and amber leaves.

No.	Source	Adjusted Sum of Squares	Adjusted Mean Squares	*F*-Value	*p*-Value	Adjusted Sum of Squares	Adjusted Mean Squares	*F*-Value	*p*-Value
			**Yellow**	**Leaves**	**Extract**		**Amber**	**Leaves**	**Extract**
Model	9	0.3904	0.0433	10.26	0.010	0.3324	0.0369	6.02	0.031
Linear	3	0.2560	0.0853	20.19	0.003	0.2506	0.0835	13.61	0.008
*X*_1_	1	0.0180	0.0180	4.27	0.094	0.0040	0.0040	0.66	0.454
*X*_2_	1	0.0300	0.0300	7.10	0.045	0.1035	0.1035	16.86	0.009
*X*_3_	1	0.2080	0.2080	49.19	0.001	0.1431	0.1431	23.31	0.005
Quadratic	3	0.0662	0.0220	5.22	0.053	0.0181	0.0060	0.99	0.470
*X*_1_·*X*_1_	1	0.0219	0.0219	5.19	0.072	0.0069	0.0069	1.13	0.336
*X*_2_·*X*_2_	1	0.0022	0.0022	0.53	0.500	0.0115	0.0115	1.88	0.229
*X*_3_·*X*_3_	1	0.0484	0.0484	11.46	0.020	0.0003	0.0003	0.05	0.831
Two-Way Interaction	3	0.0681	0.0227	5.37	0.051	0.0636	0.0212	3.46	0.108
*X*_1_·*X*_2_	1	0.0225	0.0225	5.32	0.069	0.0552	0.0552	9.00	0.030
*X*_1_·*X*_3_	1	0.0400	0.0400	9.46	0.028	0.0020	0.0020	0.33	0.591
*X*_2_·*X*_3_	1	0.0056	0.0056	1.33	0.301	0.006400	0.006400	1.04	0.354
Error	5	0.0211	0.0042			0.030692	0.006138		
Lack-of-Fit	3	0.0186	0.0062	5.05	0.170	0.014225	0.004742	0.58	0.684
Pure Error	2	0.0024	0.0012			0.016467	0.008233		
Total	14	0.4115				0.363173			

**Table 5:** The analysis of variance for the α-tocopherol relative amount in *P. persica* yellow leaves and amber leaves.

No.	Source	Adjusted Sum of Squares	Adjusted Mean Squares	*F*-Value	*p*-Value	Adjusted Sum of Squares	Adjusted Mean Squares	*F*-Value	*p*-Value
			**Yellow**	**Leaves**	**Extract**		**Amber**	**Leaves**	**Extract**
Model	9	5328.77	592.09	67.21	0.000	80,477.0	8941.9	64.90	0.000
Linear	3	4864.83	1621.61	184.08	0.000	73,642.6	24,547.5	178.16	0.000
*X*_1_	1	74.60	74.60	8.47	0.033	995.9	995.9	7.23	0.043
*X*_2_	1	4783.40	4783.40	543.00	0.000	72,603.4	72,603.4	526.93	0.000
*X*_3_	1	6.83	6.83	0.77	0.419	43.3	43.3	0.31	0.599
Quadratic	3	422.92	140.97	16.00	0.005	6028.1	2009.4	14.58	0.007
*X1·X1*	1	113.78	113.78	12.92	0.016	1913.2	1913.2	13.89	0.014
*X2·X2*	1	248.04	248.04	28.16	0.003	3373.6	3373.6	24.48	0.004
*X3·X3*	1	28.56	28.56	3.24	0.132	278.1	278.1	2.02	0.215
Two-Way Interaction	3	41.03	13.68	1.55	0.311	806.2	268.7	1.95	0.240
*X1·X2*	1	2.50	2.50	0.28	0.617	16.7	16.7	0.12	0.742
*X1·X3*	1	25.86	25.86	2.94	0.147	645.9	645.9	4.69	0.083
*X2·X3*	1	12.67	12.67	1.44	0.284	143.6	143.6	1.04	0.354
Error	5	44.05	8.81			688.9	137.8		
Lack-of-Fit	3	41.62	13.87	11.43	0.082	674.1	224.7	30.37	0.032
Pure Error	2	2.43	1.21			14.8	7.4		
Total	14	5372.82				81,165.9			

**Table 6:** Coded variables and their levels for the RSM.

Independent Variable	Low (−1)	Level Middle (0)	High (1)
Pressure (MPa), *X*_1_	10	20	30
Temperature (°C), *X*_2_	40	50	60
CO_2_ flow rate (kg·h^−1^), *X*_3_	1	2	3

## Data Availability

Not applicable.

## Sample Availability

Samples of the extracts are available from the author.
